# Genomewide analysis of circular RNA in pituitaries of normal and heat-stressed sows

**DOI:** 10.1186/s12864-019-6377-7

**Published:** 2019-12-23

**Authors:** Haojie Zhang, Baoyu Hu, Jiali Xiong, Ting Chen, Qianyun Xi, Junyi Luo, Qingyan Jiang, Jiajie Sun, Yongliang Zhang

**Affiliations:** Guangdong Province Key Laboratory of Animal Nutritional Regulation, National Engineering Research Center for Breeding Swine Industry, College of Animal Science, South China Agricultural University, Wushan Road, Tianhe District, Guangzhou, Guangdong 510642 People’s Republic of China

**Keywords:** Hyperthermia, Noncoding RNA, Pituitary, Profiling, Sows

## Abstract

**Background:**

As a newly characterized type of noncoding RNA, circular RNA (circRNA) has been shown to have functions in diverse biological processes of animals. It has been reported that several noncoding RNAs may regulate animals’ response to heat stress which can be easily induced by hyperthermia in summer. However, the expression and functions of circRNAs in the pituitary of sows and whether they participate in heat stress adaption are still unclear.

**Results:**

In this study, we found that high temperature over the thermoneutral zone of sows during the summer increased the serum heat shock protein 70 (HSP70) level, decreased the superoxide dismutase (SOD) vitality and prolactin (PRL) concentration, and induced heat stress in sows. Then, we explored circRNA in the pituitary of heat-stressed and normal sows using RNA sequencing and bioinformatics analysis. In total, 12,035 circRNAs were detected, with 59 circRNAs differentially expressed, including 42 up-regulated and 17 down-regulated circRNAs in pituitaries of the heat-stressed sows. Six randomly selected circRNAs were identified through reverse transcription PCR followed by DNA sequencing and other 7 randomly selected differentially expressed circRNAs were verified by quantitative real-time PCR analysis. The predicted target genes regulated by circRNAs through sponging microRNAs (miRNAs) were enriched in metabolic pathway. Furthermore, the predicted circRNA–miRNA–mRNA interactions showed that some circRNAs might sponge miRNAs to regulate pituitary-specific genes and heat shock protein family members, indicating circRNA’s roles in pituitary hormone secretion and heat stress response.

**Conclusions:**

Our results provided a meaningful reference to understand the functions of circRNA in the porcine pituitary and the mechanisms by which circRNA may participate in animals’ response to heat stress.

## Background

Circular RNA (circRNA) is a class of noncoding RNA (ncRNA), and numerous circRNAs are formed by head-to-tail splicing of exons [1] during transcription. CircRNAs were first observed in eukaryotes [2], but they were usually considered as useless byproducts [3] with low abundance and uncertain functions. Recently, with the rapid development of high-throughput sequencing technologies and bioinformatics, circRNAs have received significant attention. Most circRNAs originate from protein-coding genes and consist of complete exons [4]. The biological functions of circRNAs are being identified, and several studies have indicated that circRNA may act as sponges of microRNA (miRNA), sequestering and competitively suppressing miRNA activity [5, 6]. Besides, some intronic RNAs mainly located in the nucleus may interact with RNA polymerase II to influence gene transcription [7]. Some circRNAs containing ribosome entry sites may be translated into proteins [8]. Up to now, knowledge about the pig circRNAs is relatively limited comparing to mice and humans. Although several circRNAs have been discovered in pig muscles [9], brains [10], and other common tissues [11], the information about circRNAs in the pig pituitary is little known.

The mammalian pituitary is an important endocrine organ that modulates the stress response, metabolic homeostasis, growth, reproduction, and lactation [12]. These critical functions of the pituitary are mainly regulated by 6 different hormones that secreted by 5 specialized cell types within the anterior pituitary [13]. Recently, various ncRNAs such as miRNA and long noncoding RNA (lncRNA) in the pituitary have been identified to participate in some biological processes [14, 15]. Circular RNA in the pituitary of sheep have been analyzed and indicated to have functions in embryo pituitary development and endocrine regulation [16]. Therefore, circRNAs may be the new regulatory factors during the endocrine regulation in pigs and other mammalian animals.

High temperature has profound effects on sows ingestion, reproduction, lactation, and metabolism [17]. Especially in the south of China, high temperatures last half a year, which easily cause heat stress and negatively influences sow health and performance. Recent studies have found the expression alteration of ncRNAs in the mammalian heat stress response and indicated that ncRNAs may be novel regulators during heat stress [18]. In homeothermic animals, such as rat and mice, heat stress altered miRNAs expression patterns in the rat small intestine [19] and increased the expression of miR-1, miR-21, and miR-24 in the mice heart to generate a cardioprotective phenotype resistant to I/R injury [20]. Heat stress may also manipulate miRNA levels by affecting the enzymes that involved in miRNAs’ biogenesis [21]. Besides, heat stress could alter lncRNAs expression. Two lncRNA species including B2 RNA in mice and Alu RNA in humans accumulate at relatively low levels during normal cellular growth, however, their abundance transiently increases by as much as 40-fold under certain conditions of heat stress [22, 23]. Since circRNAs are also non-coding RNAs and could function as a type of ceRNA capable of sequestering miRNA activity, it is reasonable to speculate that circRNAs may vary their expression and play a role in response to heat stress. In addition, it has reported that heat stress could affect circRNAs expression and biogenesis in plants [24, 25]. However, little attention has been focused on circRNAs’ alteration and function in animals during heat stress. In this study, we researched the circRNA expression profiles in the anterior pituitaries of sows obtained in winter, when the temperature was in the thermoneutral zone, and summer, when the temperature was high. The results of our study may help to better understand the roles of circRNAs in the regulation of the pituitary function and the response to heat stress.

## Methods

### Animals and experiment design

This study used 12 healthy sows (Landrace), which were purchased from the Guangzhou thoroughbred farm (Guangzhou, Guangdong, P. R. China). Six sows were obtained during the winter months of 2017, with a moderate average temperature (19.6 ± 0.41 °C), and designated the thermoneutral (TN) group. Another 6 sows were obtained during the summer months of 2017, with a high average temperature (30.2 ± 0.40 °C), and designated the heat stress (HS) group. The body weight (in the range of 250 ± 10 kg) and parity (between 5 and 7) were balanced in the 2 test groups. The sows consume the same diet in summer and winter months.

### Sample collection

Incubate the pig with an endotracheal tube (30 cm length, 8 mm ID) and anesthetize pig with isoflurane (4.5% of tidal volume by mask) [26]. Then, the pigs were euthanized by exsanguination under a surgical plane of the isoflurane anesthesia [27]. The anterior pituitaries were carefully dissected and stored at − 80 °C until further processing. The blood samples were collected in clean tubes and centrifuged at 3000×g for 20 min at 4 °C after a room temperature clotting. The supernatant of the blood was separated and stored at − 30 °C for preservation. All animal experimentation complied with the laboratory animal management and welfare regulations approved by Standing Committee of Guangdong People’s Congress (Guangzhou), China.

### Serum assays

The activity of serum total superoxide dismutase (SOD) was measured by a SOD assay kit following the instructions of the manufacturer. The levels of serum heat shock protein 70 (HSP70), cortisol, and prolactin (PRL) were quantified using a HSP70 ELISA assay kit, cortisol ELISA assay kit, and PRL ELISA assay kit, respectively, following the instructions of the manufacturer. Nanjing Jiancheng Bioengineering Institute (Nanjing, P. R. China) provided all the commercial assay kits.

### Total RNA extraction and sequencing

Three pituitary samples of each group, including the TN group and the HS group, were selected. Following the protocol of the manufacturer, we used Trizol Reagent (Invitrogen, Carlsbad, CA) to isolate total RNA of the 6 samples. The RNA quantity and quality were assessed using an RNA 6000 Nano Lab-Chip Kit and Agilent 2100 Bioanalyzer (Agilent Technologies, Inc., Santa Clara, CA) with an RNA integrity number > 7.0. Then, ribosomal RNA was depleted from the total RNA following the instructions of the Epicentre Ribo-Zero Magnetic Gold Kit (Illumina, Inc.). After the depletion of rRNA, the remaining RNAs were digested with RNase R (Epicentre, Madison, WI) at 37 °C for 10 min and further were reverse transcribed to construct the cDNA library using the mRNA-Seq Sample Preparation Kit (Illumina, Inc). Finally, the Illumina HisSeq 4000 platform were used to sequence the libraries with 150-bp paired-end reads at LC Sciences in Hangzhou, P. R. China.

### Circular RNA identification and differential expression analysis

Firstly, raw reads were filtered using Cutadapt [28] by removing the adaptor contaminating reads and low-quality reads. Then, the remaining reads were multimapped to the Sscrofa10.2 genome using Bowtie 2 and TopHat2 [29, 30]. Extract the unmapped reads and continue to use TopHat-Fusion [31] to map the genome. The mapped reads were first reassembled to circRNA using CIRCexplorer2 [32, 33]; then, both the TopHat-Fusion and CIRCexplorer2 were used to identify the back-splicing reads in unmapped reads. Finally, a circRNA can be confirmed if it has at least 1 back-splicing read. The expression level of circRNA was quantified as fragments per kilobase of transcripts per million mapped reads (FPKM) using StringTie [34]. Only circRNA with 2 or more back-splicing reads were kept. Significantly differentially expressed circRNA were identified with the *P*-value < 0.05 and |log2 (fold change)| ≥0.585 between the two groups.

### PCR analysis, DNA sequencing, and quantitative real-time PCR validation

Total RNA was extracted from anterior pituitaries of the sows using Trizol Reagent (Invitrogen). Then, the PrimeScript RT Reagent Kit with gDNA Eraser (Takara, Dalian, P. R. China) was used to synthesize cDNA for circRNA. Polymerase chain reaction was conducted using specific primers for different circRNA. The circRNA primers sequences were provided in Additional file 1: Table S1. We used agarose gel electrophoresis and DNA sequencing to confirm the PCR products. Furthermore, the PCR products sequences, the pig reference genome and RNA sequencing data were compared by the software DNAMAN (Lynnon Biosoft, San Ramon, CA). Polymerase chain reaction was done using the designed primers and cDNA template. The PCR conditions were 94 °C denaturation for 5 min, 40 cycles at 94 °C for 10 s, 54 to 60 °C for 15 s, and 72 °C for 30 s. We randomly chose 7 differentially expressed circRNAs and used qRT-PCR to confirm the results of RNA-seq. The qRT-PCR was conducted on a Bio-Rad CFX96 Real-Time Detection System (Bio-Rad Laboratories, Inc., Hercules, CA) with the GoTaq qPCR Master Mix (Promega, Madison, WI, USA). The relative expression levels of circRNA were calculated using the 2^−ΔΔCt^ method. Porcine GAPDH was used as an internal control. All the quantitative PCR reactions were assayed with 6 biological replicates. The primers of qRT-PCR were also provided in Additional file 1: Table S1.

### Construction of predicted competing endogenous RNA networks and enrichment analysis

In order to explore circRNAs’ sponge function of miRNAs, putative interaction targets between the miRNAs and the differentially expressed circRNAs or the mRNAs were predicted by miRanda [35] and RNAhybrid [36]. Only alignments with energies less than − 20 kcal/mol and no mismatch in the seed region were retained for further analysis. There were three steps to construct the competing endogenous RNA interaction networks. First, differentially expressed circRNAs in heat stress and all pig miRNAs and mRNAs candidates were chose. The sequences of all pig miRNAs and mRNA transcripts were respectively obtained from miRbase (ftp://mirbase.org/pub/mirbase/22.1/, accessed October 2018) and UCSC (http://genome.ucsc.edu/cgi-bin/hgTables). Second, circRNA-miRNA and miRNA-mRNA negative interactions were predicted by miRanda and RNAhybrid analyses, and the common target interactions obtained by the two analyses were retained. Third, the potential interactions between circRNAs and miRNAs or mRNAs were established and the visualized circRNA-miRNA-mRNA network was constructed by Cytoscape 3.5.1 [37]. Besides, the probability (based on the shared miRNAs) for each ceRNA pair was calculated according to the previous report [38–40].

For enrichment analysis, we used DAVID software [41] to make Gene Ontology (GO) analysis and KEGG pathway enrichment of the genes that predicted to be regulated by the differentially expressed circRNAs. GO terms and KEGG pathways for which *P* < 0.05 were considered significantly enriched.

### Statistical analysis

The temperature and humidity of the swine pen in the winter and summer months were recorded every day in this study. The average daily temperature and humidity were determined using the recording data. The formula: THI = [(1.8 × T) + 32] − [0.55 × (1 − RH)] × (1.8 × T − 26) was used to calculate the average THI (temperature humidity index), in which T and RH respectively represent the air temperature in degrees Celsius and the relative humidity in percent [42]. Statistical analyses were performed by the SPSS software. Differences between groups were determined using an independent 2-sample t-test and considered statistically significant at *P* < 0.05. All the data are presented as the means ± standard error.

## Results

### Environmental indicators and the effect of high temperature on serum hormones and the antioxidant index

**Fig. 1:**
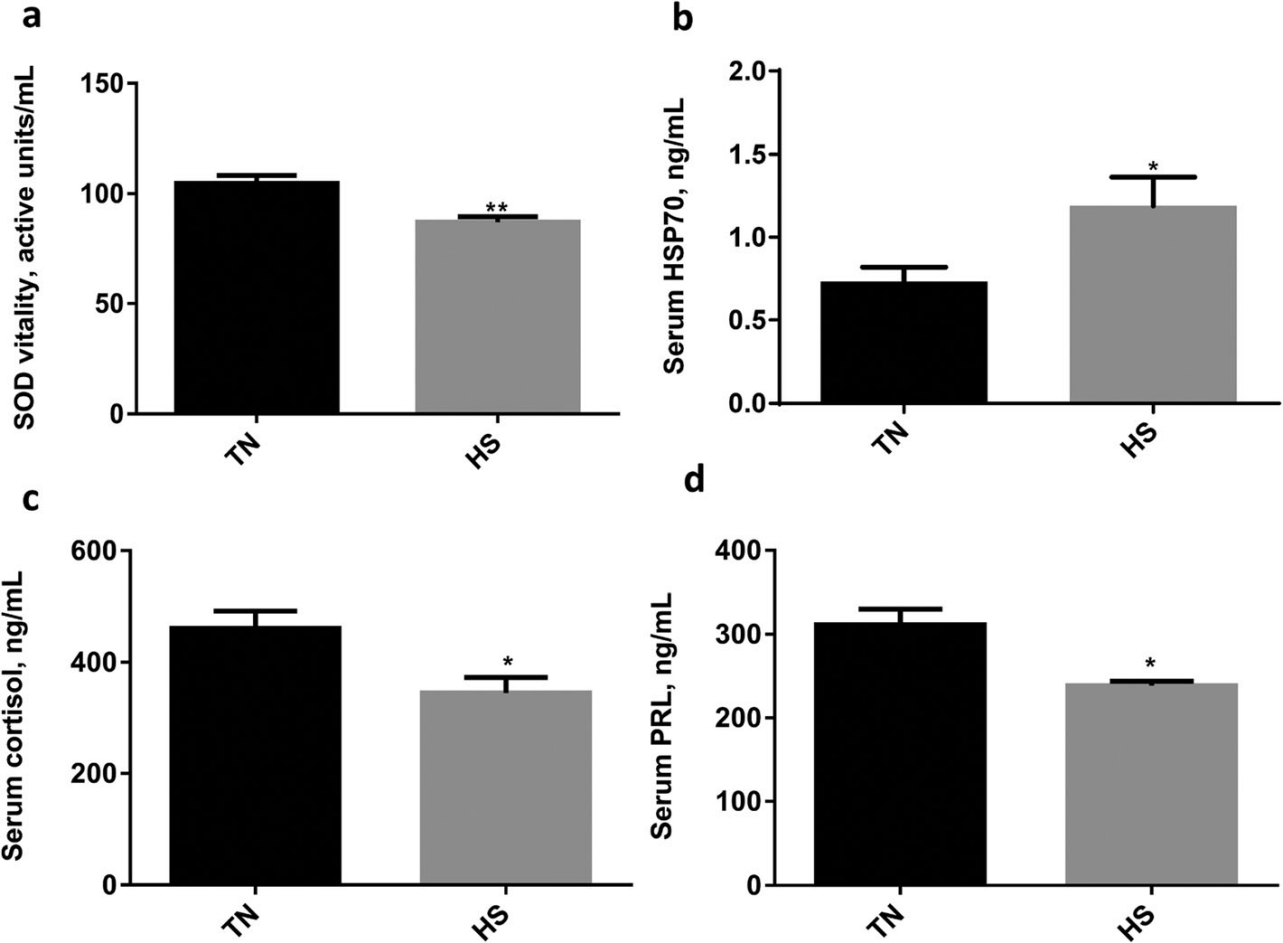
The influence of heat stress on hormones and the antioxidative index. **a** The superoxide dismutase (SOD) vitality of sow serum in the thermoneutral (TN) and heat stress (HS) groups and (**b**–**d**) the level of heat shock protein 70 (HSP70), cortisol, and prolactin (PRL) of sow serum in the TN and HS groups. Values are expressed as means and SEM. *n* = 6; *P < 0.05; **P < 0.01 (t-test)

The average temperature and humidity were 19.6 ± 0.41 °C and 67.2% ± 0.01, respectively, from December 2016 to January 2017, and the average temperature and humidity were 30.2 ± 0.40 °C and 75.3% ± 0.02, respectively, from June 2017 to July 2017. This data was used to calculate the THI, which characterizes the environmental conditions of the pen. The THI during winter was about 65.6 and was 82.5 during summer, which was well above 72. We then measured the alteration of serum HSP70, PRL, cortisol, and SOD vitality in the two groups. The SOD vitality of serum in the HS group was lower comparing with the TN group (Fig. 1a). At the same time, the level of the heat stress–sensitive protein HSP70 increased significantly in the HS group (Fig. 1b). We also found that high temperature reduced the level of cortisol (Fig. 1c) and PRL (Fig. 1d) in the HS group. The results above indicated that sows obtained in summer suffered from heat stress.

### Abundance and characteristics of circular RNA in the sow pituitary

**Fig. 2:**
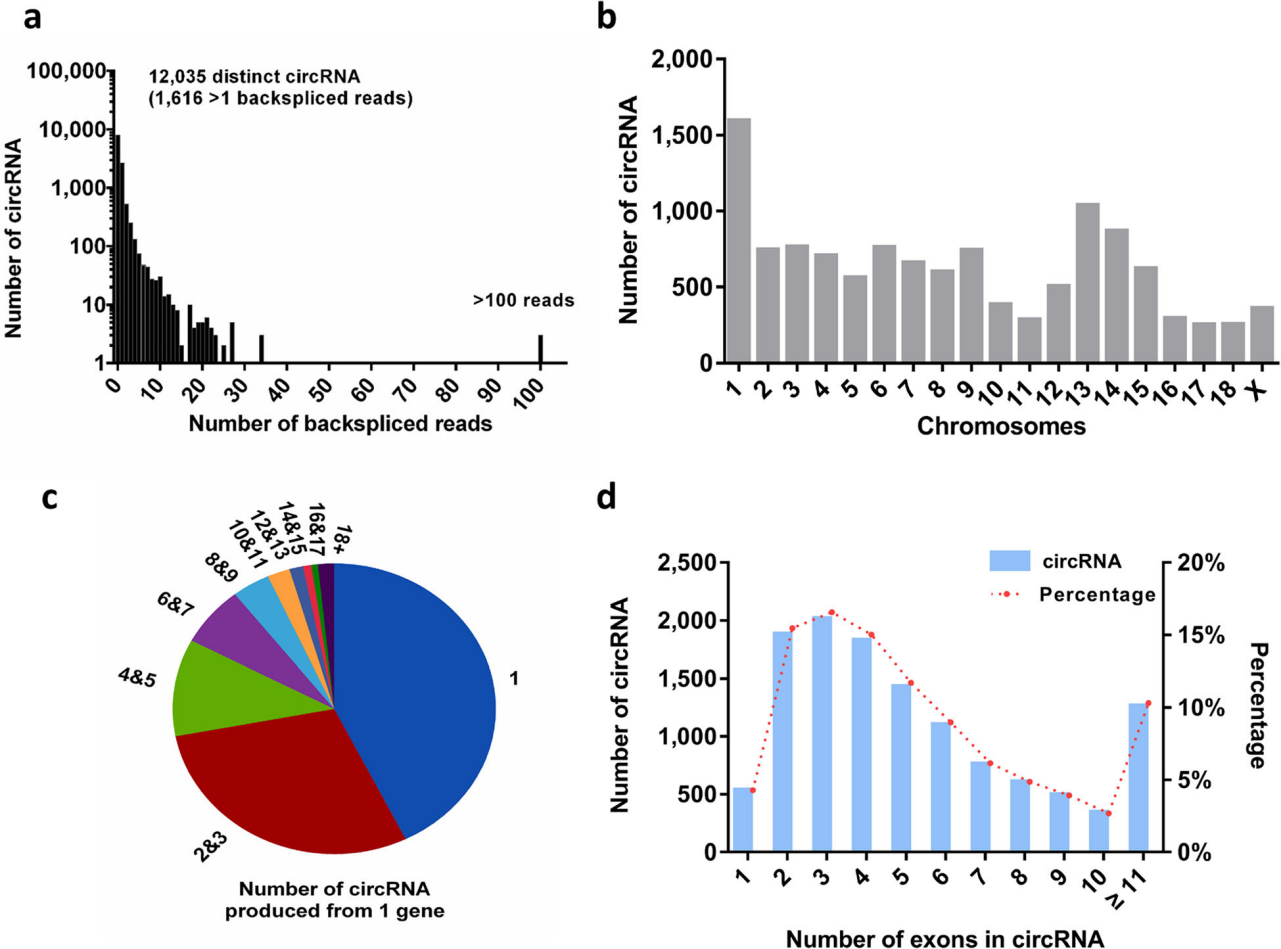
Molecular characteristics of the sow circular RNA (circRNA). **a** The number of circRNA and back-spliced reads identified in the pituitaries of the thermoneutral (TN) and heat stress (HS) groups. **b** Distribution of the circRNA among pig chromosomes of 6 pituitaries. **c** Number of circRNAs produced from 1 gene (12,035 circRNAs from 3577 host genes). **d** Distribution of the exon number of circRNA in the 6 pituitaries of the sows

In order to explore the circRNA expression profiles of the sow pituitary under heat stress, we used RNA-seq analyses to characterize the circRNA from 6 normal anterior pituitaries of the sows obtained during warm seasons (TN group) and hot seasons (HS group). From these 2 data sets, a total of 12,035 unique circRNAs were detected and 1616 of these candidates had at least 2 head-to-tail splicing reads (Fig. 2a). Meanwhile, 3 circRNAs (circRNA2294, circRNA1646 and circRNA2869) contained more than 100 back-splice reads in all the samples. For all pituitary samples, the detected circRNAs located in all the pig chromosomes and the chromosome 1 had the largest number of circRNAs (Fig. 2b). The host genes of the detected circRNAs were also analyzed and we found that multiple circRNAs could derive from a single gene (Fig. 2c). A striking example is that CHD2 may generate 53 distinct circRNAs. In addition, we further examined the number of circRNA exons and discovered that more than 95% of circRNAs are composed of multiple exons whereas only 4.3% are a single exon (Fig. 2d). The detailed information about the total detected circRNA was presented in Additional file 2: Table S2 and Additional file 11.

### Identification of circular RNA in the sow pituitary

**Fig. 3:**
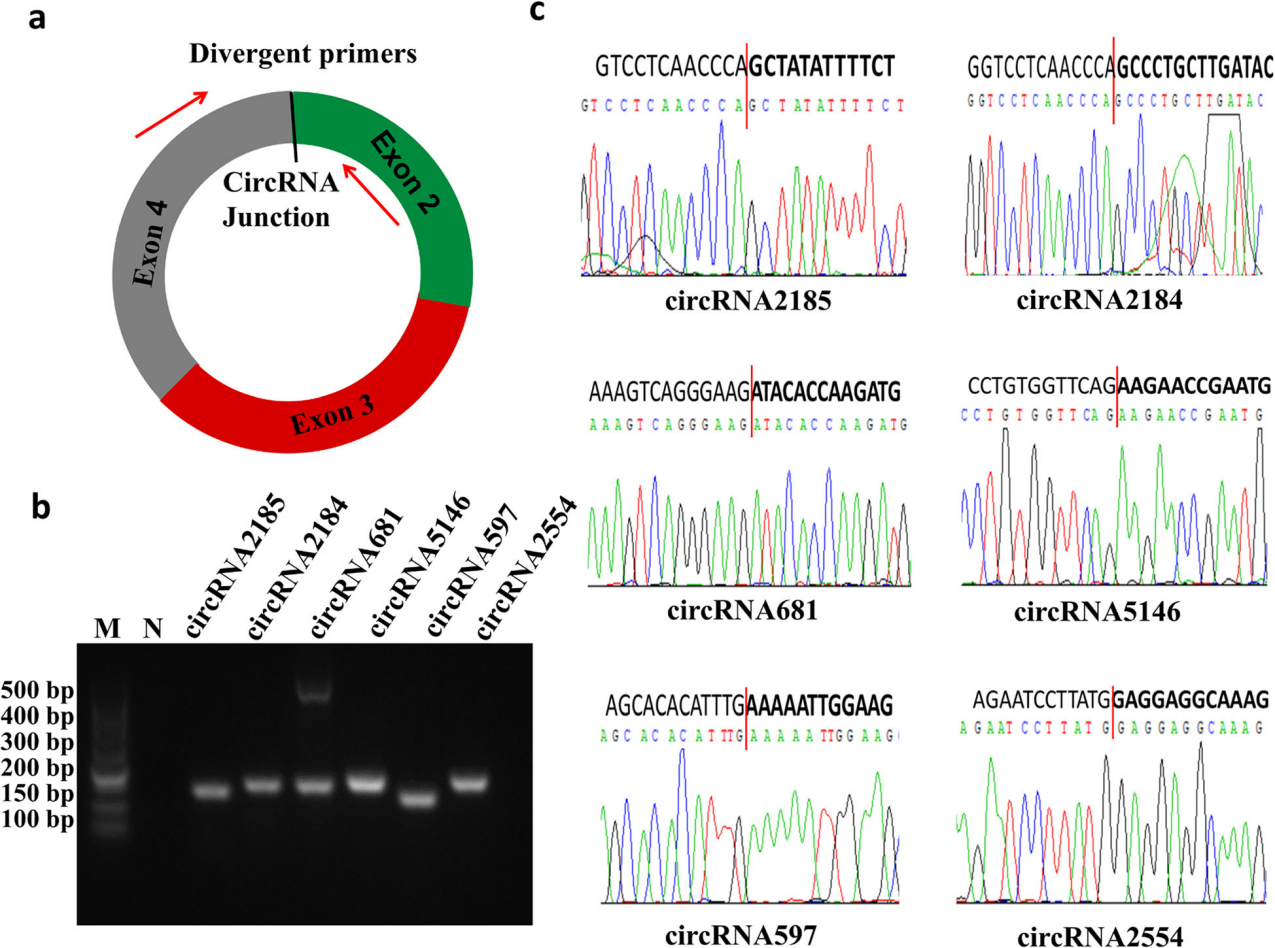
Verification of circular RNA (circRNA) data from RNA sequencing. **a** Divergent primers to amplify the circular junctions. Red arrows represent divergent primers. **b** Reverse transcription PCR amplification of circRNA with divergent primers. The PCR products of circRNA2185, circRNA2184, circRNA681, circRNA5146, circRNA597, and circRNA2554 were analyzed using gel electrophoresis. M is the marker and N is the negative control. **c** Head-to-tail splice junctions were confirmed using DNA sequencing

In order to identify the detected circRNAs in the RNA-seq results, 6 circRNAs were randomly selected and amplified by PCR with the designed divergent primers (Fig. 3a). The gel electrophoresis results showed that each circRNA had a single band at the expected location (Fig. 3b). Then, the DNA sequencing results were used to compare with the normal DNA sequences to confirm the back-splicing junctions (Fig. 3c). Finally, we successfully identified these six circular RNAs.

### Analysis and validation of differentially expressed circular RNA

**Fig. 4:**
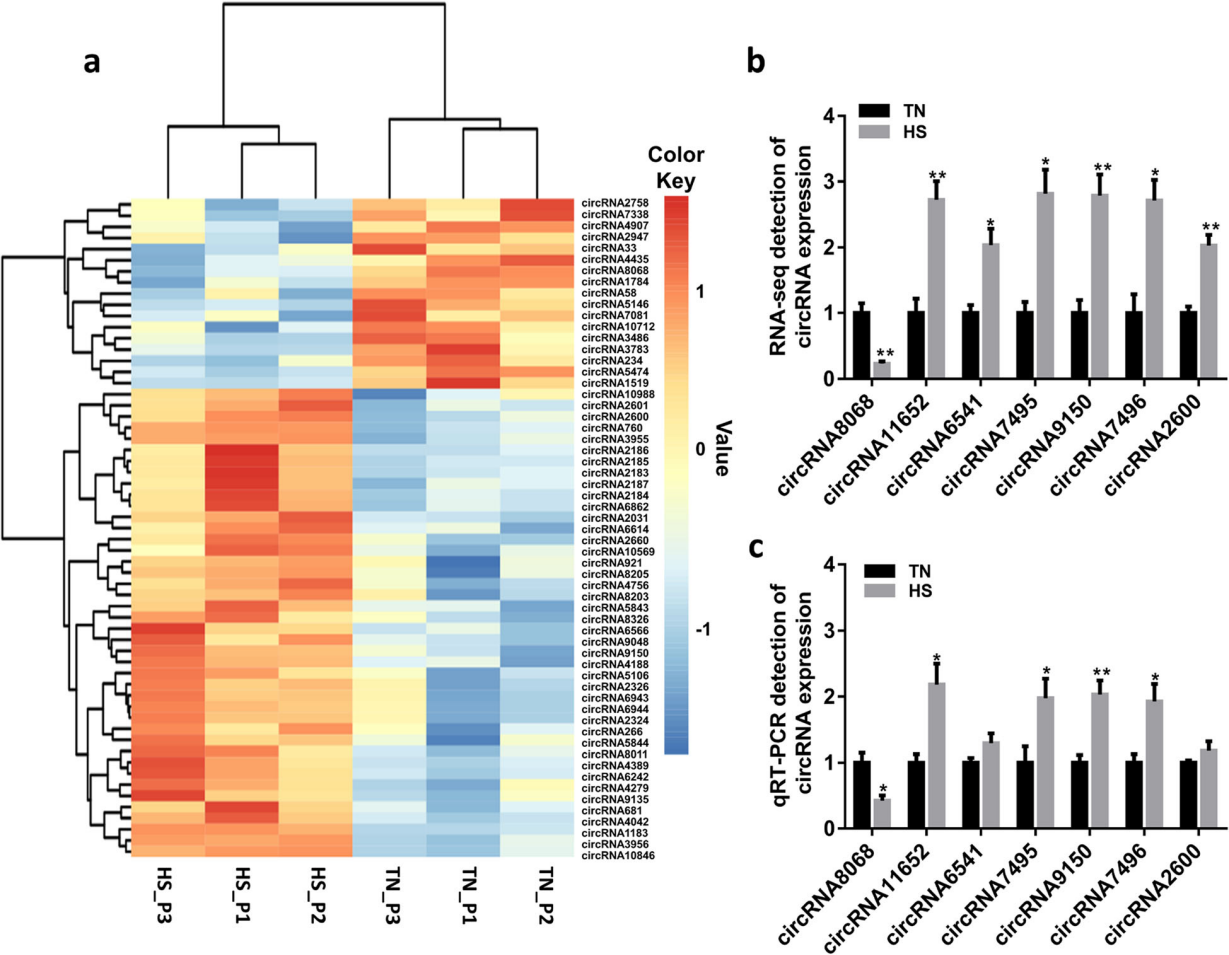
Analysis and validation of differentially expressed circular RNA (circRNA). **a** The heat map of the circRNA that were differentially expressed between the thermoneutral (TN) group and the heat stress (HS) group. Blue represents low expression, and orange represents high expression (*P* < 0.05). **b** Expression of differentially expressed circRNA was determined with data from RNA sequencing (RNA-seq; 3 samples each group). **c** Expression of differentially expressed circRNA was determined using quantitative real-time PCR (qRT-PCR; 6 samples each group). Values are expressed as means and SEM. *P < 0.05; **P < 0.01 (t-test). HS_P1 = heat stress_pituitary 1; HS_P2 = heat stress_pituitary 2; HS_P3 = heat stress_pituitary 3; TN_P1 = thermoneutral _pituitary 1; TN_P2 = thermoneutral _pituitary 2; TN_P3 = thermoneutral _pituitary 3

We used FPKM as the metric for the circRNA expression level. Only circRNAs with 2 or more back-splice reads within single samples were kept. The differentially expressed circRNA were screened with the |log_2_ (fold change)| ≥0.585 and *P* < 0.05. Thus, we identified 59 differentially expressed circRNAs in the sow pituitary between the TN and HS groups (Additional file 3: Table S3). Among them, there were 42 up-regulated and 17 down-regulated circRNAs (Fig. 4a). Then, other 7 circRNAs were randomly chosen for qRT-PCR validation. The results showed the expression of circRNA8068 significantly decreased in the HS group, and the expressions of other selected circRNAs significantly increased in the HS group (*P* < 0.05), except circRNA6541 and circRNA2600, which were highly expressed in the HS group, but has no significant difference (*P* > 0.05). The results showed a consistency between qRT-PCR and deep sequencing analysis (Fig. 4b), suggesting that the identified circRNA had a true differential expression in vivo.

### Target miRNA or gene prediction of differentially expressed circRNAs and enrichment analysis

**Fig. 5:**
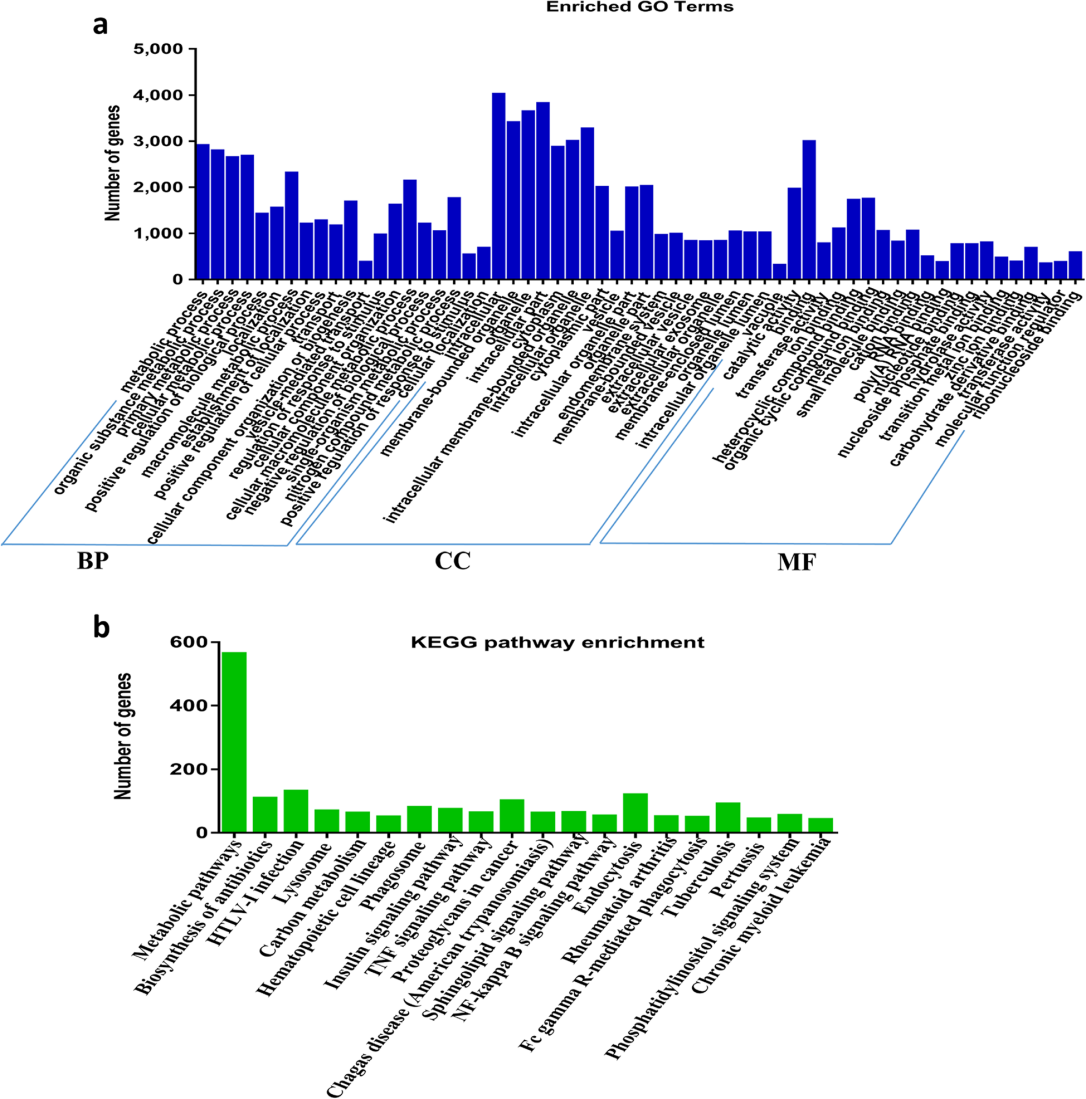
Annotations and enrichment of the predicted genes regulated by differentially expressed circular RNA. **a** Gene Ontology (GO) analysis showed significantly enriched terms (*P* < 0.05) in the categories of biological process, molecular function, and cellular components. **b** Kyoto Encyclopedia of Genes and Genomes (KEGG) pathway analysis identified the enriched terms in the HS and TN groups. BP = biological process; CC = cellular component; MF = molecular function

Circular RNAs were proved to have ‘sponge’ functions on miRNAs and further indirectly regulate mRNAs expression [1, 5]. Here, we predicted the potential interactions between all pig miRNAs and pig mRNAs or the differentially expressed circRNAs according to the miRanda and RNAhybrid pipeline. In the constructed potential circRNA–miRNA–mRNA associations, there were 110 miRNAs interacted with 10,598 mRNA transcripts and 51 differentially expressed circRNAs (Additional file 4: Table S4). In order to learn the potential functions of the differentially expressed circRNAs in the pituitary, we used GO and KEGG pathway enrichment analyses to analyze enrichment of the 10,598 mRNA transcripts. In the text, only the top 20 GO categories of three differential ontologies (Fig. 5a) and the top 20 enriched pathways from the KEGG enrichment analysis are shown (Fig. 5b). The results showed that the enriched GO terms were mainly associated with metabolic process, organic substance metabolic process, cellular metabolic process, and regulation of response to stimulus (*P* < 0.05, Additional file 5: Table S5). In addition, the KEGG pathways were mainly enriched in metabolic pathways, carbon metabolism, insulin signaling pathway and TNF signaling pathway (*P* < 0.05, Additional file 6: Table S6). Which may also indicate that circRNAs have functions in metabolic process.

### CeRNA networks construction and conservation analysis

**Fig. 6:**
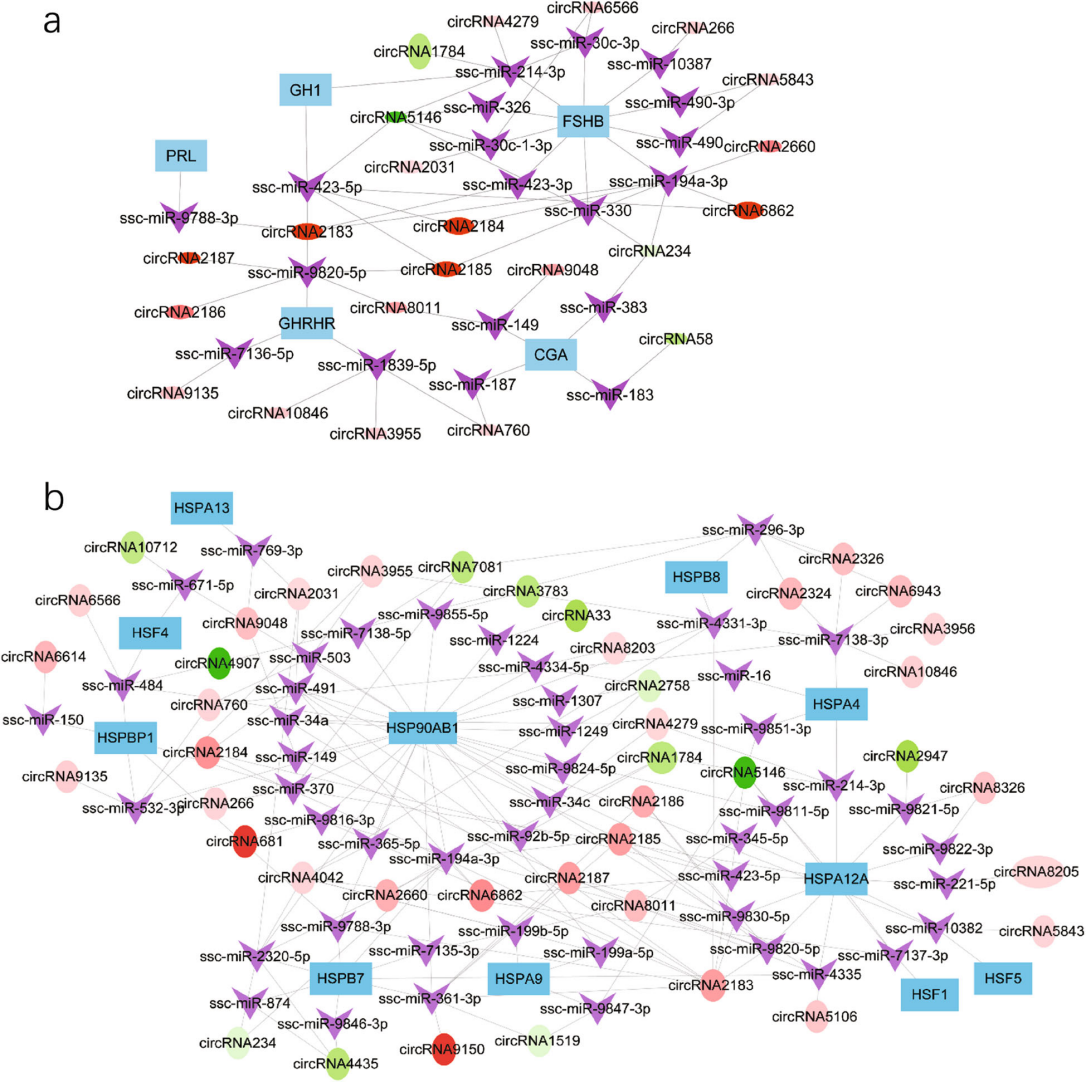
Circular RNA (circRNA)–microRNA (miRNA)–mRNA interaction networks. **a** The competing endogenous RNA (ceRNA) network of circRNA, miRNA, and pituitary-specific genes. **b** The ceRNA network of circRNA, miRNA, and heat shock protein (HSP) family members. Note: Circular nodes represent circRNA, V-type nodes represent miRNA, and square nodes represent mRNA. Red nodes represent upregulated circRNA, and green nodes represent downregulated circRNA. The size of each circular node represents the expression level of each gene in the sows, and a deeper color represents a greater difference in level between the 2 groups

Since pig pituitary circRNAs differentially expressed under heat stress, we further focused on the pituitary functional genes as well as the heat stress regulated genes, screened effective information from the previous circRNA-miRNA-mRNA network (Additional file 4: Table S4) and established 2 networks which had potential functions in regulating pituitary (Fig. 6a and Additional file 7: Table S7-A) and heat stress (Fig. 6b and Additional file 7: Table S7-B). In the networks, one node represented one gene, the edge connected by 2 genes represented a tight regulatory relationship. In Fig. 6, one network showed that 22 circRNAs could sponge 19 miRNAs to regulate 5 pituitary-specific genes including follicle-stimulating beta polypeptide (FSHB), PRL, growth hormone 1(GH1), growth hormone releasing hormone receptor (GHRHR), and Chromogranin A CGA (Fig. 6a). Another network indicated that 42 circRNAs could sponge 48 miRNAs to regulate several 11 members of the HSP family, including HSF1, HSF4, HSF5, HSPA4, HSPA9, HSPB7, HSPB8, HSP13, HSPA12A, HSP90AB1 and HSPBP1(Fig. 6b). Taken together, based on the analysis of differentially expressed circRNAs in pig pituitary under heat stress, we found that 42 ceRNA pairs (Additional file 8: Table S8-A) may regulate pituitary-specific genes and 154 ceRNA pairs (Additional file 8: Table S8-B) may be involved in heat stress.

Our study also searched pig orthologous circRNAs from human or mouse. We compared pig circRNAs to human and mouse circRNAs with clear one to one orthologs (http://asia.ensembl.org/biomart/martview/) [11]. We found 68.51% of pig pituitary circRNAs have human orthologs, whereas 54.94% of pig pituitary circRNAs have mouse orthologs (Additional file 9: Table S9-A and Table S9-B). Then, we performed sequence blast analysis of the potentially conserved pig pituitary circRNAs with human or mouse circRNAs from the datasets in circbase (http://www.circbase.org/). Blast analysis suggested that 4903 (4903/12035 = 40.74%) and 1732 (1732/12035 = 14.39%) pig pituitary circRNAs have orthologs in humans and mice respectively (Additional file 9: Table S9-C and Table S9-D). Finally, we looked for the conserved ceRNA pairs related to pituitary specific genes or heat stress genes by checking for the conservation of miRNA-target interactions (Additional file 10: Table S10-(A-C)) from humans and mice. We identified 10 ceRNA interactions in pig pituitary were conserved with humans (Additional file 10: Table S10-D). Whereas there were no conserved ceRNA interactions related to pituitary specific genes and heat stress among pigs and mice.

## Discussion

During the summer, high temperature exceeds the upper limit of the thermoneutral zone will induce heat stress in the sows. For lactating sows, this thermoneutral zone ranges from 12 to 22 °C [43]. Therefore, the sows suffered from heat stress most of the time during our experimental period. Also, the THI is an index that can be used for evaluating if the animals are under heat stress, and a THI range of 72 to 89 is considered to be moderate heat stress [44]. In our study, the calculated THI above 72 during summer months could also be an indicator of heat stress. It has been reported that heat stress reduced animal antioxidant activity [45] and altered the hormone levels of the sows [46]. The SOD vitality was reduced in the HS group, indicating the sows’ impaired antioxidant activity under heat stress. HSP70 is a universal marker of heat stress, and its expression rapidly increased when animals were subjected to heat stress [47, 48]. Similarly, the serum HSP70 level in the HS group also increased significantly relative to TN group in our study (Fig. 1b). In addition, the serum PRL level of the sow was significantly reduced under high temperature (Fig. 1d), which is accordance with former researches [49]. It is widely believed that the heat or other climatic stress can activate the hypothalamic–pituitary–adrenal axis and accompanying with an increased level of glucocorticoid (mainly cortisol) in the serum [50]. However, there was a decline in the serum cortisol level in our study (Fig. 1c). It has been reported that cortisol levels increased in the early stage of heat stress and then decreased as the heat stress continued [51]. The different alteration of cortisol concentrations may be depended on whether the heat stress is acute or chronic [52]. In this study, sows were in chronic heat stress during the summer months. As cortisol is the hormone that regulate thermogenesis, the decreased cortisol level in heat stress is benefit for reducing the metabolic heat production [50].

Previous RNA sequencing results indicated that humans [53, 54], mice [55, 56] and rats [57, 58] have abundant circRNAs. Recently, circRNAs have also been identified in several pig tissues [10, 11]. But there is no information about circRNAs in the pig pituitary. So our study was the first to explore the circRNA expression profile of the pig pituitary, and we identified 12,035 circRNAs in the pig pituitary using high-throughput sequencing. Similarly, 10,226 circRNAs were identified in sheep pituitary, and some of these were involved in hormone secretion regulation [16]. Therefore, the abundance of circRNA in pig pituitary may also indicate that circRNAs have crucial functions in the pig pituitary.

High environmental temperature during summer seasons makes sows easily subjected to heat stress, which further induces a decrease in food intake, milk yield, and reproductive efficiency as well as an alteration of endocrine status [17, 59, 60]. Studies have reported that circRNAs have functions during heat stress in plants [24]. While, the role of circRNA in mammalian animals is little known. Therefore, we analyzed the circRNA expression changes in the sow pituitary between normal and heat stress conditions. It has been reported that heat stress alters the circRNA size and the exons numbers in Arabidopsis species [24]. In our study, we did not observe these obvious alterations except the different expression level of circRNA, indicating that natural high temperature may not affect circRNA biogenesis in animals. Based on the bioinformatics analysis, we identified 59 circRNAs that were differentially expressed between the 2 groups. Circular RNA can, in theory, regulate cellular stress both positively and negatively [61]. For instance, circFOXO3 promotes cardiac senescence under oxidative stress and serum starvation [62]. Circ-RasGEF1B regulates the antimicrobial response [63]. So, we speculated that circRNAs may be the new regulators of the heat stress response.

Researches have reported that circRNA can sponge miRNA to further regulate gene expression. For an instance, CDR1as/ciRS-7 and Sry performed the sponge function in human and mouse brain development, respectively [1, 5]. In order to explore the 59 differentially expressed circRNAs’ function as ceRNA on gene regulation, we predicted target miRNA of the screened circRNA using miRanda and RNAhybrid, and created a ceRNA network containing differentially expressed circRNAs, previously reported pig miRNAs, and their target mRNAs. From the circRNA–miRNA interaction, we found that various circRNAs interact with different pituitary-related miRNAs (Additional file 4: Table S4), such as miR-128 [64], miR-132 [65], miR-16 [65, 66], miR-361-3p [67], and let-7c [68]. In addition, we also observed that a single circRNA could sponge several different miRNAs with various target sites. For example, circRNA5146 contained potential binding sites for 17 pig miRNAs (Additional file 4: Table S4), which may indicate its strong role in sponging miRNAs.

The pituitary is an important endocrine organ modulating animal growth, development, metabolism, and sexual function through a variety of pituitary hormones [69]. There were 32 pituitary-specific genes that had been reported in the Tissue Specific Genes Database (TiSGeD) database [70]. In our study, after analyzing all the circRNA–miRNA–gene interactions, we were surprised to discover that several differentially expressed circRNAs could regulate 5 of the pituitary- specific genes through miRNAs (Fig. 6a). The results indicated that these circRNAs may play important roles in secretion of hormones, including FSHB, GH1, and PRL, which could be a possible regulatory mechanism of the endocrine status alteration during heat stress. Also, it is notable that circRNA2183 was predicted to regulate GH1 and PRL through miR-423-5p and miR-9788-3p, respectively, and regulate FSHB through miR-423-3p and miR-194a-3p. CircRNA5146 was predicted to regulate GH1 through miR-423-5p and miR-214-3p, and FSHB through miR-30c-1-3p, miR-30c-3p, miR-326, miR-330 and miR-423-5p. Therefore, circRNA2183 and circRNA5146, as potential ceRNAs, may be central regulators of pituitary gland function. Since many investigations pointed out that the HSP and HSF are the key players in animals’ heat stress response [71]. We also found that the circRNAs expressed differentially between the TN and HS group could regulate HSP and HSF by interacting with miRNAs (Fig. 6b). So the circRNA-mediated network may contribute to the response and adaptation to heat stress. Certainly, all the potential interplay among genes, miRNAs, and circRNAs need further experimental analysis.

Studies have reported that some circRNAs are evolutionarily conserved among humans and mice [72, 73]. In our study, we found several pig pituitary circRNAs were also conserved with humans and mice (Additional file 9: Table S9). We identified 68.51% of pig pituitary circRNAs have human orthologs, whereas 54.94% of pig pituitary circRNAs have mouse orthologs with one-to-one orthologs analysis. A previous research has also reported that circRNAs on pig nine organs (not include pituitary) are modestly conserved with humans and mice [11]. However, our circRNAs results of one-to-one orthologs analysis were relatively higher than that of previous report, which may be related to tissue specificity. For the conservation analysis of ceRNA interactions, we identified 10 ceRNA interactions in pig pituitary were conserved with humans (Additional file 10: Table S10-D). Whereas there were no conserved ceRNA interactions related to pituitary specific genes or heat stress among pigs and mice.

To explore the function of differentially expressed circRNAs, the interrelated genes within the circRNA-mediated network (Additional file 4: Table S4) were imported to the GO Consortium and KEGG pathways for annotation analysis. The predicted genes that would be regulated by the differentially expressed circRNAs between the HS group and the TN group were enriched in GO terms associated with the metabolic process, organic substance metabolic process, cellular metabolic process, and regulation of response to stimulus. In addition, the metabolic pathways were enriched in the KEGG pathway analysis. An impact of circRNA on metabolic pathway would be of great interest because high temperature widely affects the metabolism of pigs [74, 75]. In summary, the results of our analysis suggested that the circRNA may participate in the metabolic regulation of pigs under heat stress.

## Conclusion

Our study is the first to analyze the circRNA expression profile in sow pituitary and identify the differentially expressed circRNAs in pituitary of sows under heat stress. These results suggested the sow pituitary has abundant circRNAs and the differentially expressed circRNAs may paly functions on hormone synthesis and stress response by regulating the specific pituitary genes and heat stress–related genes. Besides, the target genes of the differentially expressed circRNAs were enriched in biological process such as metabolic process and regulation of response to stimulus. Therefore, our study provided a reference for investigating the functions of circRNAs in pituitary regulation and heat stress response.

## Supplementary information

**Additional file 1: Table S1.** Primer sequence for circRNAs and housekeeping genes.

**Additional file 2: Table S2.** The information of total detected circRNAs.

**Additional file 3: Table S3.** The information of differentially expressed circRNAs.

**Additional file 4: Table S4.** The total interaction relationships among miRNAs, mRNAs and the differentially expressed circRNAs.

**Additional file 5: Table S5.** GO annotation enrichment analysis.

**Additional file 6: Table S6.** KEGG pathway enrichment analysis.

**Additional file 7: Table S7**. A. The interactions among miRNAs, pituitary specific genes and the differentially expressed circRNAs. B. The interactions among miRNAs, heat stress related genes and the differentially expressed circRNAs.

**Additional file 8: Table S8.** A. The *p*-value of each ceRNA interactions related to pituitary specific genes. B. The p-value of each ceRNA interactions related to heat stress genes.

**Additional file 9: Table S9**. A. Orthologous circRNAs among pigs and humans with one to one orthologs analysis. B. Orthologous circRNAs among pigs and mice with one to one orthologs analysis. C. Orthologous circRNAs among pigs and humans with blast analysis. D. Orthologous circRNAs among pigs and mice with blast analysis.

**Additional file 10: Table S10**. A. The interactions of miRNA- pituitary specific genes or heat stress related genes and miRNA-differentially expressed circRNAs in pig pituitary. B. Conversed miRNA-gene and miRNA-circRNA interactions in humans. C. Conversed miRNA-gene and miRNA-circRNA interactions in mice. D. Conversed ceRNA interactions among pigs and humans.

**Additional file 11.** The sequences of total detected circRNAs.

## Data Availability

The datasets generated and/or analyzed during the current study are available in the NCBI Sequence Read Archive (SRA) repository with the accessions SRP199497, BioProject accession number PRJNA544777. Additional datasets supporting the conclusions of this article are included within the manuscript and its additional files.

## Abbreviations

ceRNA: Competing endogenous RNA
circRNA: circular RNA
GO: Gene Ontology
HSP70: Heat shock protein 70
KEGG: Kyoto Encyclopedia of Genes and Genomes
miRNA: microRNA
ncRNA: noncoding RNA
PRL: prolactin
qRT-PCR: Quantitative real time PCR
SOD: Superoxide dismutase
THI: Temperature humidity index
